# Comparative nutritional characteristics of the three major Chinese *Dendrobium* species with different growth years

**DOI:** 10.1371/journal.pone.0222666

**Published:** 2019-09-20

**Authors:** Yingdan Yuan, Maoyun Yu, Bo Zhang, Xin Liu, Jinchi Zhang

**Affiliations:** 1 Co-Innovation Center for Sustainable Forestry in Southern China, Nanjing Forestry University, Nanjing, Jiangsu, China; 2 Jiangsu Province Key Laboratory of Soil and Water Conservation and Ecological Restoration, Nanjing Forestry University, Nanjing, Jiangsu, China; 3 Anhui Tongjisheng Biotechnology Co., Ltd, Lu'an, Anhui, China; 4 Cultivation and Industrialization Center of Rare Medicinal Plants in Ta-pieh Mountains, West Anhui University, Lu'an, Anhui, China; 5 Department of Biology, University of Miami, Coral Gables, FL, United States of America; National University of Kaohsiung, TAIWAN

## Abstract

*Dendrobium*, an important medicinal plant, is a source of widely used herbal medicine to nourish the stomach and treat throat inflammation. The present study is aimed at distinguishing and evaluating three major *Dendrobium* species by comparing physiochemical characteristics and understanding differences between different growth years in the Ta-pieh Mountains. Polysaccharides and total alkaloids of *Dendrobium* were determined, and the amino acids and trace elements were determined by UPLC (Ultra High-Performance Liquid Chromatography) and ICP-MS (Inductively coupled plasma mass spectrometry). It can be seen from the results that the polysaccharide content of these three kinds of *Dendrobium* in different growth years ranges from 249.31 mg·g^-1^ to 547.66 mg·g^-1^, and the highest content is in the 3-year-old *Dendrobium huoshanense*. The total alkaloid content ranges from 0.21 mg·g^-1^ to 0.54 mg·g^-1^, and the highest content is also the 3-year-old *Dendrobium huoshanense*. We determined the amino acid content of these three *Dendrobium* in different growth years, and we can see that each of the three kinds of *Dendrobium* contain seven kinds of amino acids required by the human body. We conducted a safety evaluation of the essential trace elements of *Dendrobium*, and the results showed that the dosage of 12g·d^-1^ *Dendrobium* prescribed in China Pharmacopoeia is in accordance with the recommended daily intake of trace elements recommended by the Food and Drug Administration of the United States, and will not cause trace element poisoning. Linear discriminant analysis was carried out on the basis of amino acids and trace elements and confirmed the applicability of multi-elemental analysis for identifying different *Dendrobium* species.

## Introduction

*Dendrobium* is an important edible-medicinal plant belonging to orchid family, with more than 1400 species in the world. In China, there are 74 species and two varieties of *Dendrobium* [1], more than 50 of which can be used as ‘Shihu’, a popular tonic and a traditional Chinese medicine [2]. *Dendrobium* is a perennial herb, which contains polysaccharides, alkaloids, essential amino acids and some trace mineral elements [3, 4]. As a high-grade drug, it had been documented in Shennong’s Herbal, as the stems of *Dendrobium* have been used to make a tonic or a functional medicine to nourish stomach, relieve throat inflammation, improve eyesight and promote body fluid production for thousands of years in Chinese medicine [5, 6]. Furthermore, Chinese people use *Dendrobium* as supplements in their daily life. In recent years, the market demand has been constantly expanding.

Essential trace elements are very important nutrients for human body functions and growth [7]. A deficiency of trace elements could bring on structural and physiological abnormalities, while high levels of those essential trace elements may be harmful to human health [8, 9]. There are many studies reporting on trace elements in medicinal herbs, such as *Panax ginseng*, *Salvia miltiorrhiza*, *Houttuynia cordata* and so on [10–13]. In *Dendrobium* species, there is one report about the determination of trace elements levels for identifying the cultivation technique of *Dendrobium officinale* [14]. In this study, we attempted to distinguish three different *Dendrobium* species using the determination of trace elements and amino acids.

**Fig 1:**
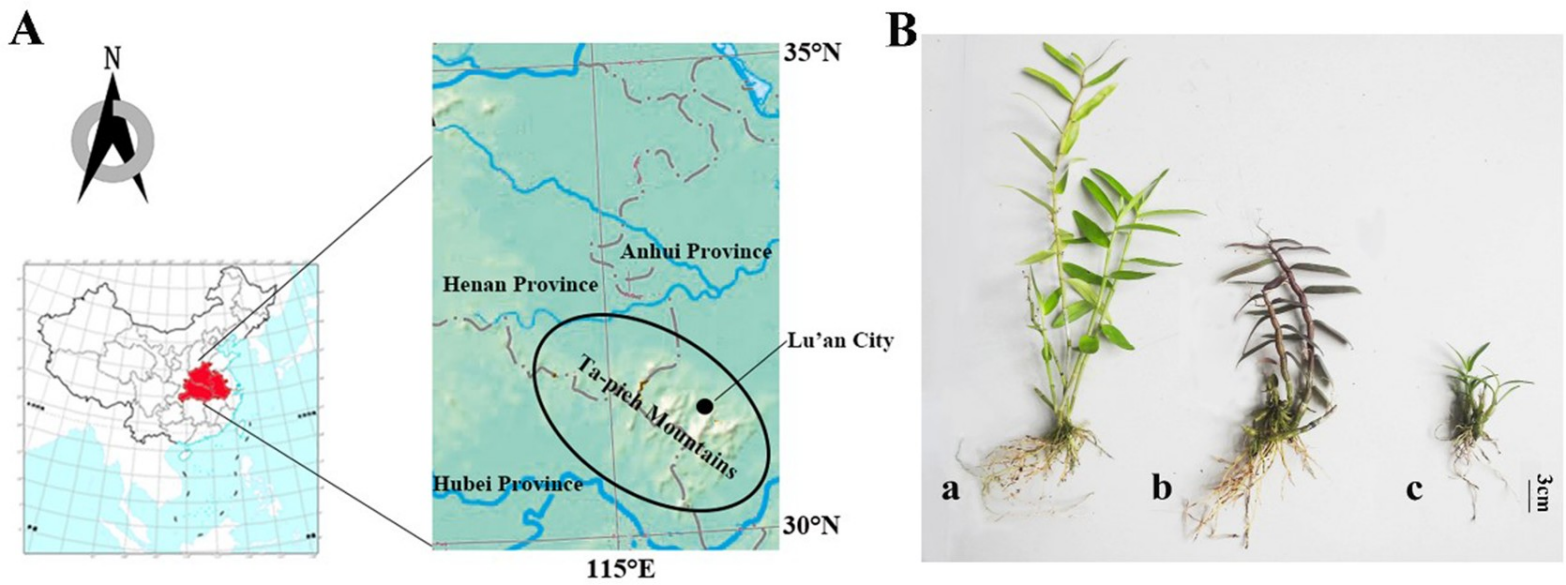
The natural habit of Dendrobium—location map of Ta-pieh Mountains area. Maps from China National Administration of Surveying, Mapping and Geoinformation (http://www.cgs.gov.cn/). (B) Samples from different Dendrobium species. (a) *Dendrobium moniliforme*. (b) *Dendrobium officinale*. (c) *Dendrobium huoshanense*.

*Dendrobium huoshanense* is named after Huoshan County of Anhui Province and is endemic to the Ta-pieh Mountains. Due to its special geographical location and climatic environment, the Ta-pieh Mountains have become the main area in which of medicinal plants of *Dendrobium* are distributed in Anhui Province [15, 16]. The Ta-pieh Mountains are located in south-eastern China, at the border of Anhui, Hubei and Henan provinces (Fig 1A). Lu'an City is in the heart of the Ta-pieh mountains, where three kinds of *Dendrobium* are mainly produced: *Dendrobium huoshanense* C. Z. Tang et S. J. Cheng, *Dendrobium officinale* Kimura et Migo and *Dendrobium moniliforme* (Linnaeus) Swartz. These three *Dendrobium* plants were declared by Lu'an Municipal Government as a Geographical Indication (GI) product. *D*. *huoshanense*, *D*. *officinale* and *D*. *moniliforme* have a high commercial value, particularly *D*. *huoshanense* [17]. Due to the extremely low germination rate of natural *Dendrobium* seeds and the scarcity of *Dendrobium* in the wild, as well as excessive collection, existing resources cannot meet the current market demand.

In the traditional Chinese medicine market, tissue-cultured *Dendrobium* have already become the major resource of pharmaceutical *Dendrobium* [18]. Plantlets were usually regenerated through protocorm-like bodies (PLBs) or direct shoot organogenesis [19]. In *Dendrobium* cultivation, different tissue culture systems for micropropagation have been established, such as protocorms [20], shoot tips [21], axenic shoot nodal segments [22], which have been used as explants. Furthermore, many other studies including plantlet strengthening culture [23] and suspension culture of PLBs derived from seed embryos [24], have also been reported. Altogether, these studies have made great progress in propagating and exploiting *Dendrobium*.

Driven by huge economic interests, many fake species referred to as “*Dendrobium*” are circulating in the market [25]. This unhealthy market-oriented process is detrimental to the industrial development of *Dendrobium* and is not conducive to the safety of its clinical applications. Hence, there is an urgent need for a comprehensive method of germplasm identification and quality control in *Dendrobium* species. At present, there is no detailed report on a comprehensive analysis of the differences among different growth stages of three major *Dendrobium* species in the Ta-pieh Mountains (Fig 1A). Because the characteristics of *D*. *huoshanense* are different from the two other *Dendrobium* species, including its long nutrient accumulation time, we chose the three-year and four-year-old *D*. *huoshanense* to compare, and we also made a comparison of three *Dendrobium* species in three years. The main aim of this study was to evaluate the medicinal value of three kinds of major *Dendrobium* species and provide information for these three kinds of *Dendrobium* in the Ta-pieh Mountains area, China on the best age classes to harvest from measurements of its amino acids (Thr, Val, Met, Leu, Ile, Phe, Lys) and trace elements (Fe, Zn, Mg, Mn, Se, Cr) by UPLC and ICP-MS.

## Materials and methods

### 2.1. Chemicals and reagents

All chemicals used in this study were of analytical grade. All the water used in the study was ultrapure, obtained from a Milli-Q Plus ultrapure purification system (Millipore, Bedford, MA, USA). The amino acids standards were purchased from National Institutes for Food and Drug Control (China). UPLC grade acetonitrile and methanol, and formic acid, were supplied from Merck (Darmstadt, Germany). Concentrated HNO_3_ (70%) was purchased from Merck (Darmstadt, Germany). The ICP-MS multi- elemental standard stock solution (10 mg·L^-1^) was obtained from Agilent (Santa Clara, USA). The mercury standard (1000 mg·L^-1^) was obtained from National Standard Material Center (China).

All glass containers were soaked in 10% HNO_3_ for 12 h, cleaned with ultrapure water and air dried before use. All plastic containers, polypropylene flasks, pipette tips, polytetrafluoroethylene digestion tubes, and reagents that came into contact with samples and standards were checked for contamination.

### 2.2 Instrumentation

All samples that needed to be dried were treated with an electric drying oven (Humgine, China). Amino acids were analyzed using Agilent 1290 Ultra High-Performance Liquid Chromatography (UPLC) (Agilent, USA), equipped with an automatic liquid sampler, a fluorescence detector and a diode array detector. Trace elements were determined by NexION 300X (PerkinElmer, USA) Inductively coupled plasma mass spectrometry (ICP-MS), equipped with a cyclonic spray chamber and a Meinhard nebulizer. The MARS microwave digestion instrument (CEM, USA) was used for acid digestion of samples. The polysaccharides and total alkaloids were measured using an ultraviolet-visible spectrophotometer (Thermo Scientific GENESYS, USA).

### 2.3 Sample collection and preparation

*Dendrobium* plants were artificially cultivated in the greenhouse of Anhui Tongjisheng Biotechnology Company, Lu'an, China. Seed germination was cultured on half-strength Murashige and Skoog (MS) medium. Seeds were germinated to form protocorms, then growth of protocorm-like bodies were cultured on half-strength MS medium containing adding 6-BA 0.1 mg·L^−1^, NAA 0.5 mg·L^−1^ and 1% additives (30 g·L^−1^ sucrose + 4 g·L^−1^ agar + 20% potato) under a 12/12 h light–dark cycle (approx. 30 μmol·m−2 S-1) at 25 ± 2°C, the seedlings were finally formed. After 18 months of age, the plants were transplanted into pots and placed in the greenhouse at a temperature of 25–27°C with a light/dark cycle of 12/12 h and 60–70% relative humidity. The planting substrate was selected to be breathable and moisturizing: bark + sawdust and other mixed fermentation (1:1), and then stone chippings added. From the grown plants of the respective seedlings of the three species of *Dendrobium*, three-year-old and four-year-old *D*. *huoshanense*, and two-year-old and three-year-old *D*. *moniliforme* and *D*. *officinale* were selected to provide 5 replicates of each sample (Fig 1B). The voucher specimens were authenticated by the authors and deposited at the College of Forestry in Nanjing Forestry University, Nanjing, China.

After removal of roots and leaves, we obtained stem samples. All stem samples were washed with pure water, placed in a 60°C and oven dried to constant weight, and then crushed and sieved for the determination.

### 2.4 Extraction procedure and analysis of polysaccharide

Polysaccharides were extracted according to the method described by Chinese Pharmacopoeia (version 2010). Initially, samples (0.3g) were precisely weighed, 200 ml of water was added, and the samples heated and refluxed for 2 h, filtered, then diluted to 250 ml. We accurately measured 5 ml of the solution into a 50 ml centrifuge tube, added 25 ml of ethanol, shook and refrigerated for 1 h, centrifuged at 4000 r∙min^-1^ for 20 min, discarded the supernatant, and centrifuged again with 20 ml of 80% ethanol (same as above). We repeated these 2 times, poured off the supernatant, and dissolved precipitate in heated water to a constant volume of 50 ml.

The polysaccharide content was determined by the phenol-sulphuric acid method [26, 27]. 1 ml of sample solution was transferred to the test tube, then 1 ml of 5% phenol solution was added. The solution was mixed thoroughly and 5 ml of concentrated sulfuric acid was added, shaken and placed in a 75°C water bath for 20 mins. Afterwards, it was cooled until it was at room temperature, the absorbance was measured at 490 nm using UV-visible spectrophotometer with 1 ml of water as a blank, and the test was performed in parallel three times. The calibration curve was prepared from the glucose reference, and the equation of regression was: Y = 7.3717X-0.0025, *R*^*2*^ = 0.9998, where Y is the absorbance and X is the concentration. Quantification of polysaccharides was performed on the basis of linear calibration plots of the absorbance versus the corresponding concentration.

### 2.5 Extraction procedure and analysis of total alkaloid

The method of Bush et al. was modified to analyze the alkaloid content [28]. Briefly, 0.5g of samples were precisely weighed, and soaked 1 ml of 25% ammonia solution for 30 mins and then extracted with 10 ml of chloroform in a 100 ml conical flask. The flasks were weighed and placed in a 75°C water bath for 2 h and then allowed to cool to room temperature. Chloroform was added to their original weight. Afterward, 2 ml of the above filtrate was mixed with 8 ml of chloroform as sample solution.

The total alkaloid content was determined using the method described as follows [29]. 2 ml of the sample solution was precisely transferred to a 15 ml centrifuge tube, diluted to 10 ml with chloroform. 5 ml of pH 4.5 buffer and 1 ml of 0.04% bromocresol green solution were added, after which the mixture was left to stand for 30 mins after strongly shaking for 3 mins. Subsequently, 1 ml of 0.01 mol·L^-1^ NaOH was added to 5 ml of the lower fractions for analysis. The alkaloid was determined by using a UV-visible spectrophotometer at 620 nm with dendrobine as a reference standard. The calibration curve was prepared from the dendrobine reference, and the equation of regression was: Y = 9.5429X-0.038, *R*^*2*^ = 0.9998, where Y is the absorbance and X is the concentration. Quantification of total alkaloids was performed on the basis of linear calibration plots of the absorbance versus the corresponding concentration.

### 2.6 Analysis of amino acids by UPLC

The amino acid test solution was obtained as follows [30]. 3g of *Dendrobium* samples were precisely weighed, ground, and put into a plug test tube. Then 10 ml of 6 mol·L^-1^ hydrochloric acid was added, and the samples were hydrolyzed in a 110° C oven for 24 h. They were taken out and let to cool to room temperature. After filtering and the filtrate was transferred to evaporating dish, evaporated in a water bath dissolved with 0.1 mol·L^-1^ of hydrochloric acid and diluted to 10 ml.

2.0 ml of test solution was transferred to a 10 ml centrifuge tube, and then 1.0 mol·L^-1^ triethylamine acetonitrile solution and 0.1 mol·L^-1^ PITC acetonitrile solution 2.0 ml were added respectively. Vortex blending was applied for 1 min and then the samples were left at room temperature for 60 mins. After adding 4.0 ml n-hexane, vortex blending was applied for 1 min and then they were left at room temperature for 10 min. We discarded the upper n-hexane layer, extracting it 3 times and removed the lower solution and placed it in 5ml volumetric flask, with 0.05 mol·L^-1^ sodium acetate solution (pH value of 6.5) to volume. The extract was then filtered by a 0.22 μm cellulose membrane syringe filter before UPLC analysis.

Chromatographic separation was carried out on a Poroshell 120 SB-C18 column (2.1 × 150 mm, 2.7 μm; Agilent Technologies), equipped with a pre-filter (porosity 2 μm, 2.1 mm) at 25°C with a flow rate of 0.25 ml·min^-1^. The mobile phase was a mixture of 0.1% formic acid–water (A) and acetonitrile (B). The injection volume was 2 μl. The gradient program of the mobile phase was as follows: 0–7 mins, 5–17% B; 7–14 min, 17–25% B; 14–16 mins, 25–28% B; 16–22 mins, 28–30% B; 22–33 mins, 30–85% B; 33–40 mins, 85–95% B. The detection wavelength was set at 254 nm. Quantification of amino acids was performed on the basis of linear calibration plots of the peak areas versus the corresponding concentration.

### 2.7 Analysis of trace elements by ICP–MS

The trace elements content of *Dendrobium* was determined using the method described as follows [14]. Powdered samples (0.500 ± 0.005g) were weighed accurately into the digestion tank, and 5.0 ml concentrated HNO_3_ was added for pre-digestion. After 1 h, 2 mL of 30% (w/w) H_2_O_2_ was added and the mixture was subjected to a microwave digestion furnace. Then we set the maximum power of 100 W, so that the temperature rose to 100°C within 10 mins, and was held at the temperature for 20 mins, and then the digestion tank cooled to room temperature. After microwave digestion, the digested solutions were diluted to 25 mL with ultra-pure water. Then the ICP-MS instrument was used to determine the contents of the samples. The optimized operating conditions of the ICP-MS used in this work are summarized in Table 1. All ICP-MS parameters were optimized to improve the sensitivity. Sensitivity was measured before each set of measurements.

**Table 1:** Optimized conditions of the ICP-MS.

ICP-MS instrument	Perkin-Elmer NexION 300D
ICP parameter	
RF power	1500W
Plasma gas flow rate	15 L·min^-1^
Auxiliary gas flow rate	1.20 L·min^-1^
Nebulizer gas flow rate	1.08 L·min^-1^
Sweeps	20
Dwell time	50 ms
Replicates	3
Reading	1
Scanning mode	Peak hopping
Sampling cone orifice (Ni)	1.2 mm
Skimmer cone orifice (Ni)	1.0 mm
Dynamic reaction cell gas flow (NH3)	0.7 mL·min^-1^
Isotopes monitored	^56^Fe, ^55^Mn, ^65^Zn, ^53^Cr, ^82^Se, ^24^Mg

### 2.8 Validation of the methods

The UPLC method for quantitation of amino acids was validated by determining the linearity, precision, repeatability, stability and recovery. The results are shown in Table 2. All of the amino acids contents showed good linearity with the determination coefficients (*R*^*2*^) ranging from 0.9992 to 0.9999 over a relatively wide concentration range. The results of precision test RSDs (relative standard deviation) of the amino acids were less than 3.47%, repeatability RSDs were less than 2.3% and stability RSDs were less than 1.76%. The overall recoveries were between 98.17 and 101.8% for the reference compounds, with RSDs less than 3.8%. Hence, the UPLC method is accurate, precise, and sensitive enough for quantitative evaluation of the amino acids in these samples.

**Table 2:** *R*^*2*^, linear range, precision, repeatability, stability and recovery of amino acids.

Amino acids	*R*^*2*^	Linear Range (mg·L^-1^)	Precision (RSD, %, n = 6)	Stability (RSD, %, n = 6)	Repeatability (RSD, %, n = 6)	Recovery (RSD, %, n = 6)	
						Mean	RSD
Threonine (Thr)	0.9999	2.916–145.8	0.93	2.3	1.2	98.46	2.6
Valine (Val)	0.9997	2.904–145.2	2.98	0.83	0.23	98.17	3.4
Methionine (Met)	0.9999	3.688–184.4	1.1	3.5	1.1	100.7	2.2
Leucine (Leu)	0.9992	3.276–163.8	1.5	2.1	0.7	100.7	3.6
Isoleucine (Ile)	0.9996	3.312–165.6	3.47	0.43	0.59	99.14	3.1
Phenylalanine (Phe)	0.9995	4.092–204.6	1.3	1.2	1.76	100.8	3.8
Lysine (Lys)	0.9998	3.692–184.6	2.93	0.34	0.63	101.8	2.6

Validation of the ICP-MS analytical method was performed using certified reference material: GBW-10016 tea (National Analysis Centre, China). Table 3 shows that the results obtained were in good agreement with certified values, which demonstrates the excellent accuracy of the selected method. The lowest concentration of the working solution for calibration use was diluted with the corresponding solvent to a series of concentrations. The limit of detection and limit of quantification for each analyte was acquired while the signal-noise ratios were respectively 3 and 10.

**Table 3:** Limits of detection and concentrations for the studied elements and standard material concentrations.

Elements	Standard material(n = 3)		Linear Range (μg·g^-1^)	Limit of detection (μg·g^−1^)	Limit of quantification (μg·g^−1^)	Recovery	
						(RSD, %, n = 6)	
	Certified levels (μg·g^-1^)	Certified levels (μg·g^-1^)				Mean	RSD
Fe	242±18	233.48	10–500	0.2	0.6	98.54	1.24
Zn	51±2	50.26	10–500	0.1	0.3	96.35	1.31
Mg	1860±110	1799.6	100–5000	1	5	98.88	1.17
Mn	500±20	499.07	10–500	0.05	0.2	98.13	0.9
Se	0.098±0.08	0.09	0.2–20	0.005	0.02	93.16	1.38
Cr	0.45±0.1	0.45	0.2–20	0.01	0.3	98.25	1.37

### 2.9 Statistical analysis

Differences in three kinds of *Dendrobium* of physicochemical characteristics were evaluated by Student's t-tests and ANOVA one-way analysis of variance, and differences were considered statistically significant at P < 0.05. Linear discriminant analysis was applied by using the Statistical Product and Service Solutions (SPSS) version 21 for windows (IBM, USA). Statistical analysis of other data (mean, standard deviation, minimum and maximum values) was done using Excel 2016 (Microsoft, USA).

## Results and discussion

### 3.1 Polysaccharide contents variation in different *Dendrobium* with different growth years

**Fig 2:**
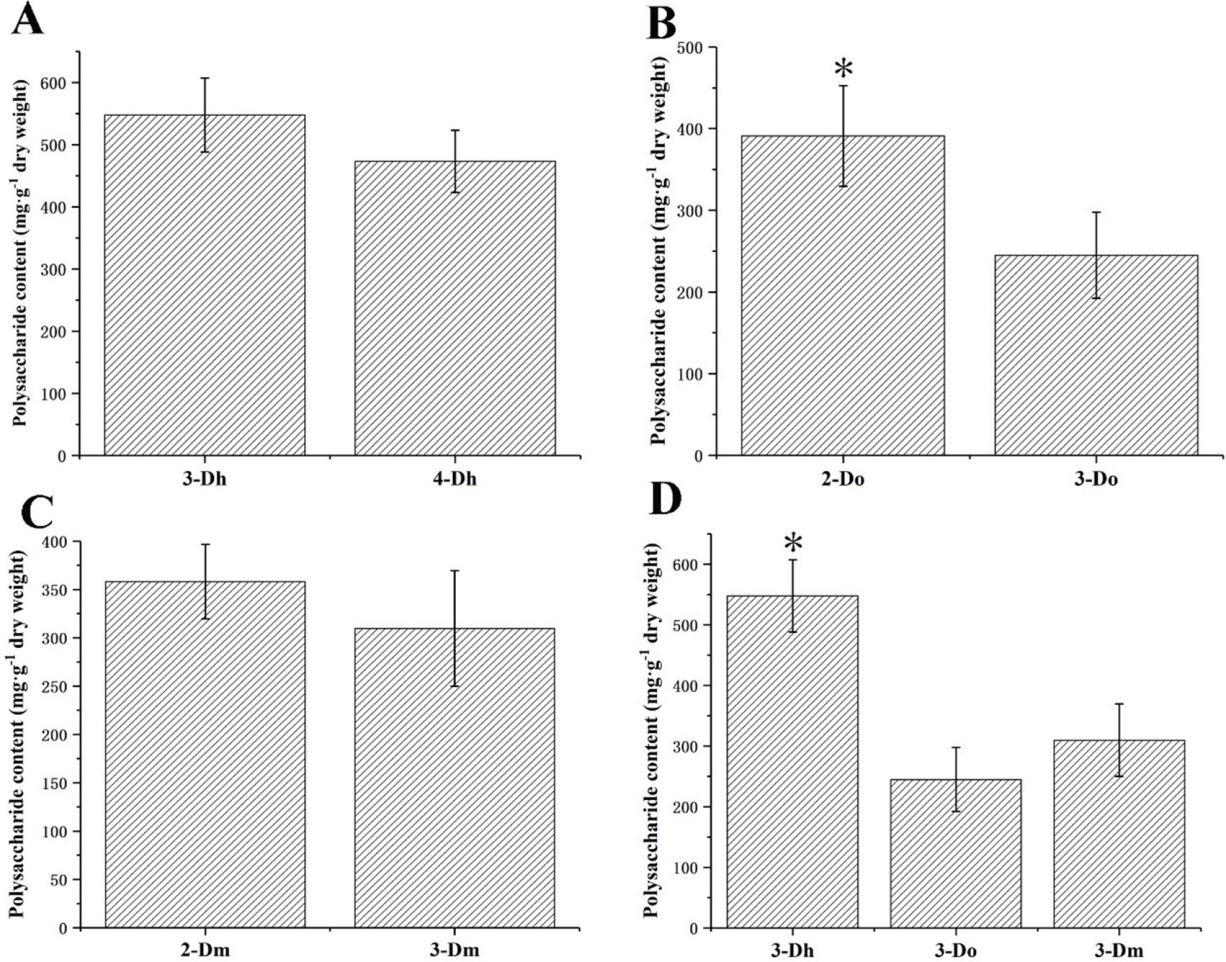
Polysaccharide contents variation in different Dendrobium with different growth years (n = 5). (A) Polysaccharide contents variation between three-year-old D. huoshanense and four-year-old *D*. *huoshanense*. (B) Polysaccharide contents variation between two-year old *D*. *officinale* and three-year-old *D*. *officinale*. (C) Polysaccharide contents variation between two-year old *D*. *moniliforme* and three-year-old *D*. *moniliforme*. (D) Polysaccharide contents variation among three different species of *Dendrobium* in three-year-old plants. 3-Dh represents three-year-old *D*. *huoshanense*; 4-Dh represents four-year-old *D*. *huoshanense*; 2-Do represents two-year-old D. officinale; 3-Do represents three-year-old *D*. *officinale*; 2-Dm represents two-year-old *D*. *moniliforme*; 3-Dm represents three-year-old *D*. *moniliforme*. When p<0.05, the difference between groups is significant, as indicated by “*”.

Polysaccharides are present naturally in food ingredients and provide many benefits to the body. It has been well documented that polysaccharides from natural sources (mushrooms, algae, lichens and higher plants) are very potent macrophage immunomodulators [31, 32]. Polysaccharide is one of the main medicinal components of *Dendrobium*. We determined the polysaccharide of *Dendrobium* in different species with different growth years. The results are shown in Fig 2. This indicates that the accumulation of *Dendrobium* polysaccharides will not be at their maximum levels if harvesting is delayed beyond a certain period of time, as the polysaccharide will be slowly consumed. According to statistical analysis, there are significant differences between the two-year-old *D*. *officinale* and the three-year-old *D*. *officinale*. At the same time, in comparing all the three-year-old *Dendrobium* species, there are also significant differences between *D*. *huoshanense* and the other two *Dendrobium* species. The average content of polysaccharides in the three-year-old *D*. *huoshanense* reached 547.66 mg·g^-1^. In view of the medicinal efficacy of polysaccharides, *D*. *huoshanense* has higher value than other two species of *Dendrobium*. However, in general, the polysaccharides of these three kinds of *Dendrobium* are in accordance with the 25.0% specified in the 2015 edition of the Chinese Pharmacopoeia.

### 3.2 Total alkaloid contents variation in different *Dendrobium* with different growth years

**Fig 3:**
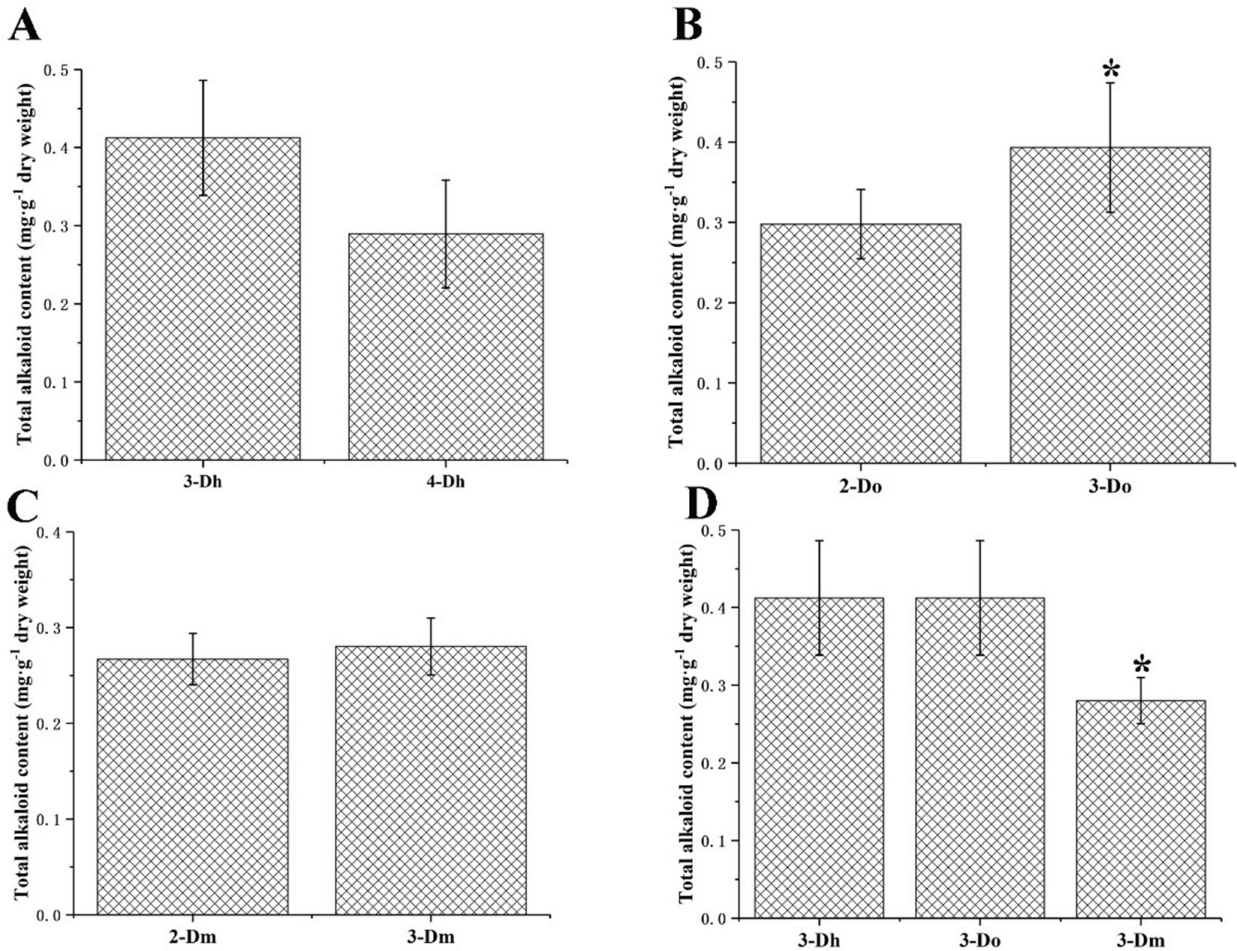
Total alkaloid contents variation in different *Dendrobium* with different growth years (n = 5). (A) Total alkaloid contents variation between three-year-old *D*. *huoshanense* and four-year-old *D*. *huoshanense*. (B) Total alkaloid contents variation between two-year old *D*. *officinale* and three-year-old *D*. *officinale*. (C) Total alkaloid contents variation between two-year old *D*. *moniliforme* and three-year-old *D*. *moniliforme*. (D) Total alkaloid contents variation among three different species of *Dendrobium* in three-year-old. When p<0.05, the difference between groups is significant, as indicated by “*”.

According to reports, alkaloids from genus *Dendrobium*, such as dendrobine, increase antioxidant capacity and show anti-cancer activity through improvement of human immunity [33]. It can be seen from the results of our measurements that the total alkaloids of the three species of *Dendrobium* are between 0.2 mg·g^-1^ and 0.4 mg·g^-1^ (Fig 3). It has also been reported in the literature that the alkaloid content of *D*. *officinale* is about 0.2 mg·g^-1^, which is consistent with our results [34]. Analysis of our results and showed that there are significant differences between the two-year-old *D*. *officinale* and the three-year-old *D*. *officinale*. Furthermore, from comparing the three-year-old *Dendrobium*, there are significant differences between *D*. *moniliforme* and the other two *Dendrobium* species. Among the three species of *Dendrobium*, the alkaloid content of *D*. *huoshanense* is still slightly higher than that of the two other *Dendrobium* species.

### 3.3 Amino acids contents variation in different *Dendrobium* with different growth years

**Fig 4:**
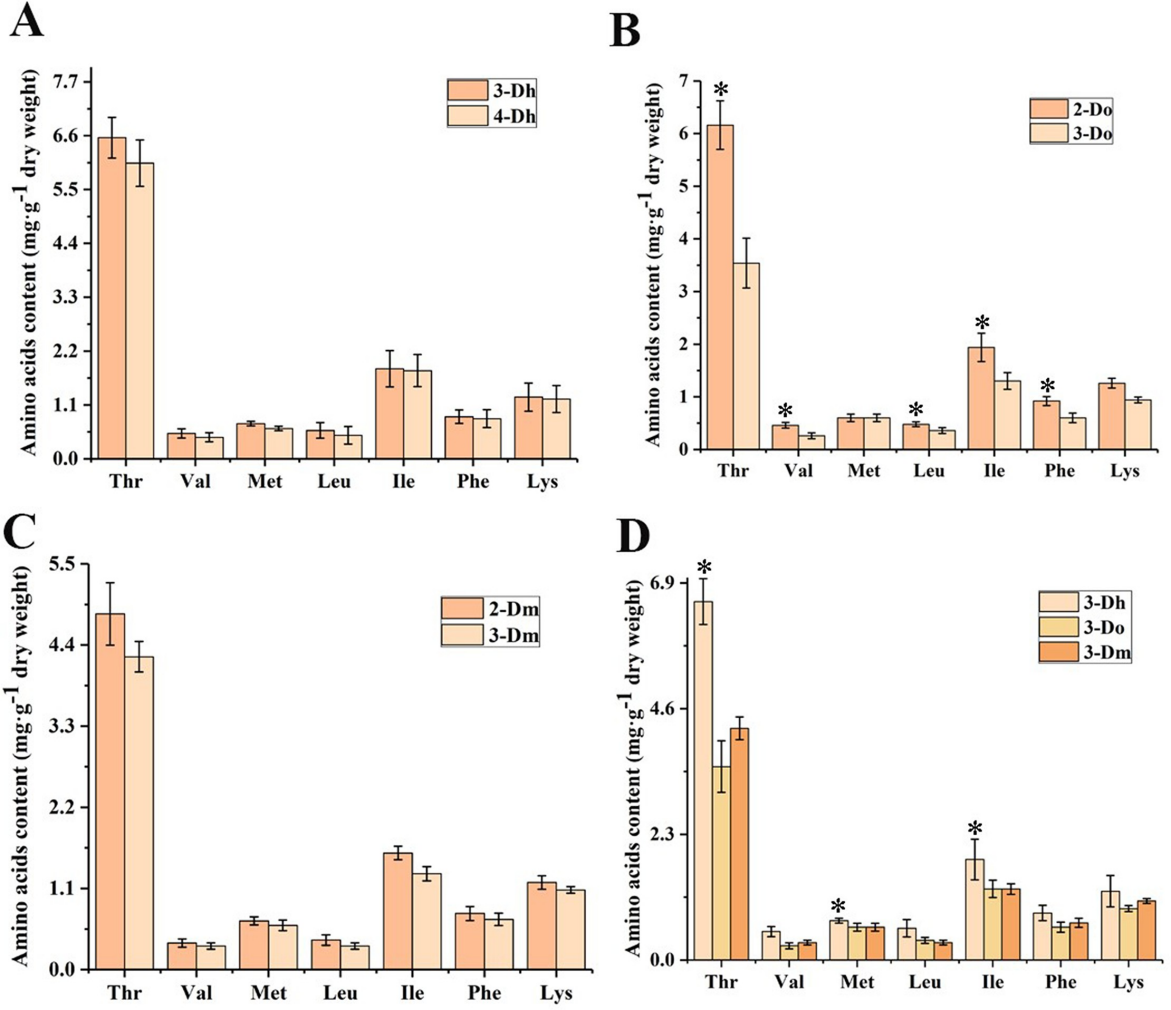
Amino acids contents variation in different *Dendrobium* with different growth years (n = 5). (A) Amino acids contents variation between three-year-old *D*. *huoshanense* and four-year-old *D*. *huoshanense*. (B) Amino acids contents variation between two-year old *D*. *officinale* and three-year-old *D*. *officinale*. (C) Amino acids contents variation between two-year old *D*. *moniliforme* and three-year-old *D*. *moniliforme*. (D) Amino acids contents variation among three different species of *Dendrobium* in three-year-old. Significant differences within the group when p<0.05, as indicated by “*”.

Amino acids have important functions in both nutrition and health [35, 36]. The human body has eight kinds of essential amino acids. Due to the instability of tryptophan, we selected for study the seven other essential amino acids in the human body. The body cannot synthesize these amino acids by itself, but they must be provided in other ways. Without these essential amino acids, the normal growth and development of the human body can be affected, leading to serious illness. Therefore, the amino acids contained in traditional Chinese medicine can be used as an auxiliary component or the main component of the treatment of certain diseases. In this study, the results of amino acids are shown in Fig 4.

Among seven essential amino acids, threonine (9.10 mg·g^-1^ in *D*. *huoshanense*) has the highest concentration, while valine (0.28 mg·g^-1^ in *D*. *huoshanense*) has the minimum value. Valine, methionine, leucine, isoleucine, phenylalanine and lysine in the three species of *Dendrobium* showed concentrations similar to the values reported by Zhang et al. [37], whereas threonine showed higher concentrations. As can be seen from Fig 4A–4C, the amino acids contents of three-year-old *D*. *huoshanense* are higher than those of four-year-old *D*. *huoshanense*. The two-year-old amino acids contents of *D*. *moniliforme* and *D*. *officinale* are higher than those of the three-year-old *D*. *moniliforme* and *D*. *officinale*. This difference may be related to factors such as the function of amino acids and the physiological and biochemical effects of *Dendrobium*. Amino acids are essential substrates for protein synthesis and play an indispensable role in plant growth and development regulation [38]. However, no previous studies have reported the differences between these essential amino acids in three species of *Dendrobium*. We compared amino acids contents of three-year-old plants of different *Dendrobium* species. As shown in Fig 4D, amino acids contents of *D*. *huoshanense* were significantly higher than the other two *Dendrobium* species. Also, by analysis of variance, threonine, methionine and isoleucine, of *D*. *huoshanense* and of the other two species of *Dendrobium* showed significant differences.

Amino acids not only play central roles as building blocks of proteins but also participate in metabolism as intermediates. Amino acid disorders can lead to clinical signs and symptoms, even serious diseases, in metabolism, immunity and the cardiovascular and nervous systems [39–42]. Among the seven essential amino acids, the content of threonine is the highest in the three *Dendrobium* species, and isoleucine and lysine are the second highest. Many studies have shown that these seven essential amino acids are contained in the *Dendrobium* species studied, but the highest values of these seven amino acids are different due to the different origins and different varieties of *Dendrobium* [37, 43].

### 3.4 Trace elements contents variation in different *Dendrobium* with different growth years

**Fig 5:**
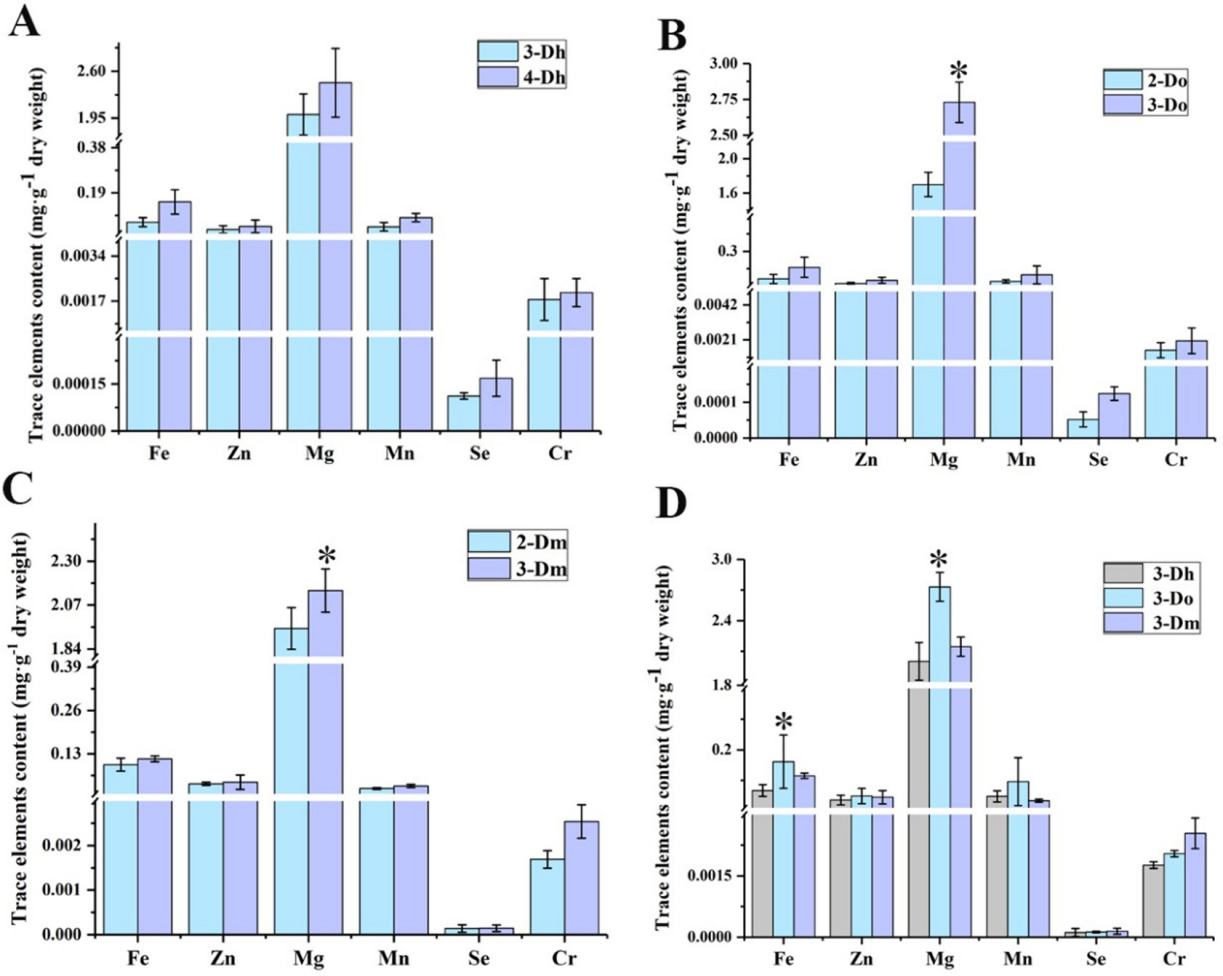
Trace elements contents variation in different *Dendrobium* with different growth years (n = 5). (A) Trace elements contents variation between three-year-old *D*. *huoshanense* and four-year-old *D*. *huoshanense*. (B) Trace elements contents variation between two-year old *D*. *officinale* and three-year-old *D*. *officinale*. (C) Trace elements contents variation between two-year-old *D*. *moniliforme* and three-year-old *D*. *moniliforme*. (D) Trace elements contents variation among three different species of *Dendrobium* in three-year-old plants. When p<0.05, the difference between groups is significant, as indicated by “*”.

The results of the elemental analysis of samples are shown in Fig 5. As shown in Fig 5A–5C, the trace element contents of four-year-old *D*. *huoshanense* are higher than those of three-year-old *D*. *huoshanense*. The trace element contents of three-year-old *D*. *moniliforme* and *D*. *officinale* are higher than those of two-year-old *D*. *moniliforme* and *D*. *officinale*. These results happen to be the opposite of the amino acids contents. Four-year-old *D*. *huoshanense* showed a high content of Zn (0.049 mg·g^-1^) and Cr (0.0018 mg·g^-1^) in all the cases. The maximum values for Fe (0.161 mg·g^-1^), Mg (2.73 mg·g^-1^) and Mn (0.096 mg·g^-1^) were high in the three-year-old *D*. *officinale*. Finally, for Se, the maximum value was 0.0016 mg·g^-1^ in three-year-old *D*. *moniliforme*. Fe, Zn, Mn, Cr and Se contents of *D*. *officinale* showed concentrations similar to the values reported by Ni et al. [14]. The results from the figures show that Fe, Zn, Mg and Mn concentrations are relatively high in *Dendrobium*, of which Mg has the highest content. Nutritionally speaking, trace elements such as Mg, Fe, Zn and Mn have concentrations greater than other trace elements in the human body. Up to now, no studies have reported the essential trace element comparisons of these three *Dendrobium* species. We compared trace elements contents of three-year-old of different *Dendrobium* species, as shown in Fig 5D. Among the three-year-old *Dendrobium*, *D*. *officinale* is higher than the other two *Dendrobium* species in Fe, Mg, Mn and Zn, this being especially significant in Mg.

Attention has been given to the harm of heavy metal elements to the human body, and the safety evaluation of essential trace elements is very necessary. A safety evaluation of the trace element level can be carried out by analyzing the dosage. Scientific recommendations were made for the dosage of the tested samples by comparing the contents of trace elements in the tested medicinal materials with the US Food and Drug Administration recommended daily intake of trace elements (RDI) (https://ods.od.nih.gov/factsheets/Iodine-HealthProfessional/) [44]. The 2010 edition of the Chinese Pharmacopoeia recorded that the daily dosage of *Dendrobium* was 6-12g dry product. According to this, we can calculate whether the elements obtained by taking 12g *Dendrobium* per day exceed the daily nutrient supply recommended by the Food and Drug Administration of the United States. Taking Fe in the three-year-old *D*. *huoshanense* as an example, the amount of Fe per day is 0.0669 mg·gg^-1^ × 12 g, which is 0.803 mg, without considering the loss of absorption. In RDI, Fe is in the range of 0.27 mg to 27 mg, and therefore can be taken safely according to the dosage prescribed by the Pharmacopoeia. The results of the calculation results are shown in Table 4. The *Dendrobium* studied in this study, when taken according to the dosage prescribed by the Pharmacopoeia, does not exceed RDI and does not causes adverse reactions or trace element poisoning.

**Table 4:** The amount of trace elements supplemented by taking *Dendrobium* 12 g·d^-1^.

Elements	3-Dh	4-Dh	2-Do	3-Do	2-Dm	3-Dm	RDI (mg)
Fe	0.802	1.828	0.732	1.932	1.171	1.379	0.27–27
Zn	0.43	0.588	0.268	0.588	0.484	0.542	2–13
Mg	24.02	29.29	20.398	32.762	23.37	25.754	30–420
Mn	0.572	1.033	0.476	1.152	0.314	0.407	0.003–2.6
Se	0.018	0.013	0.0006	0.0015	0.0017	0.019	0.015–0.07
Cr	0.02	0.0222	0.0178	0.0221	0.0202	0.0218	0.002–0.45

3-Dh: three-year-old *Dendrobium huoshanense*; 4-Dh: four-year-old *Dendrobium huoshanese*; 2-Do: two-year-old *Dendrobium officinale*; 3-Do: three-year-old *Dendrobium officinale*; 2-Dm: two-year-old *Dendrobium monilifome*; 3-Dm: three-year-old *Dendrobium moniliforme*. RDI: Recommend daily intake.

### 3.5 Linear discriminant analysis

Linear discriminant analysis (LDA) is a classical statistical approach for dimensionality reduction and classification. It can maximize the variance between groups and minimize the variance within the same group. For achieving better classification and identification of different *Dendrobium* species, LDA was carried out on the basis of 13 elements. The stepwise discriminant procedure was carried out to extract the best discriminant variable separating different *Dendrobium* species, which enters or removes variables by analyzing their effects on the discrimination of the groups based on the Wilks’ lambda criterion.

**Fig 6:**
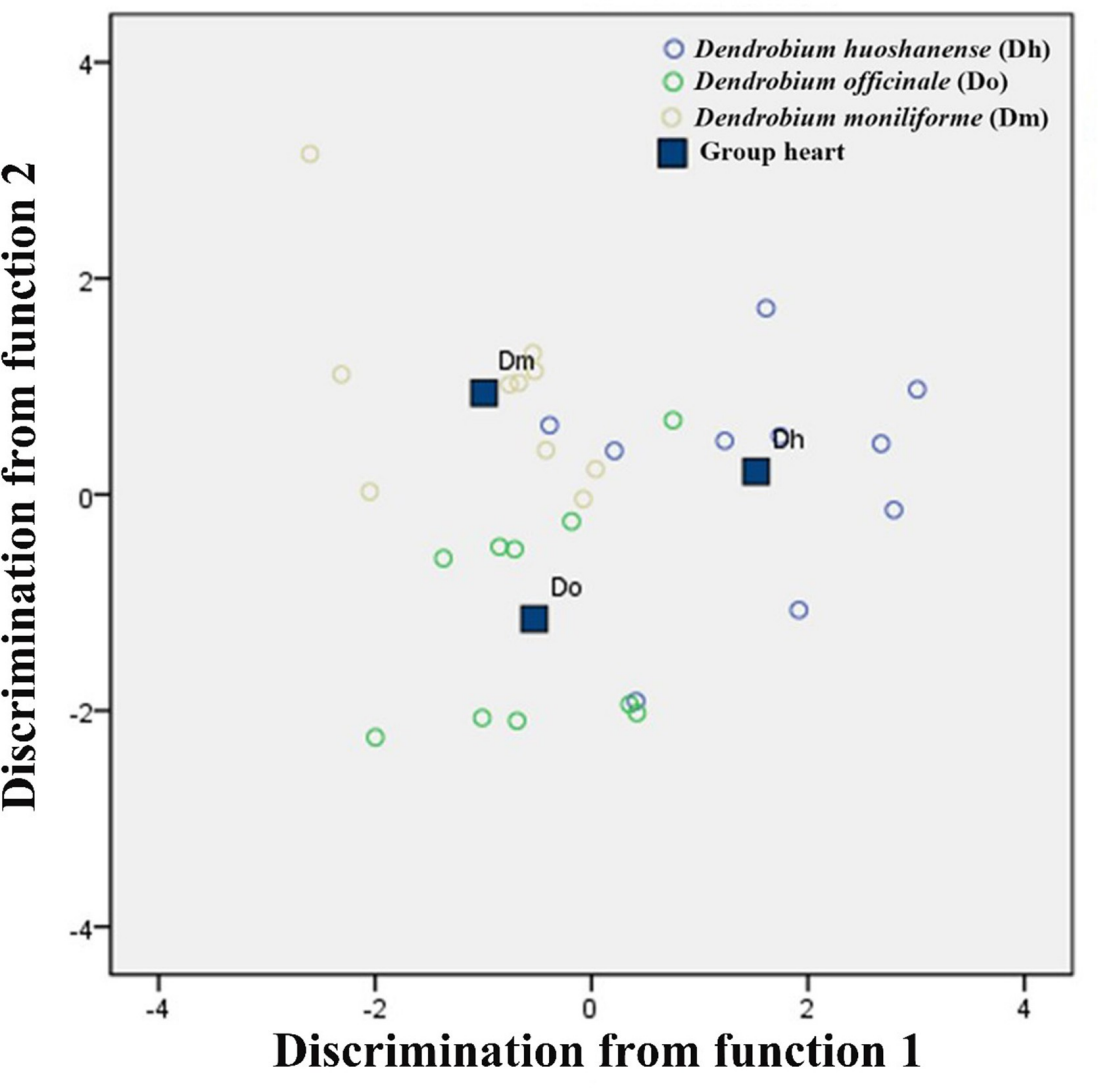
Linear discriminant analysis plot for 15 *Dendrobium* samples based on the concentrations of trace elements and amino acids. Purple circle: *Dendrobium huoshanense*; green circle: *Dendrobium officinale*; yellow circle: *Dendrobium moniliforme*.

The separation of different groups in the discriminant space was obtained by plotting the first two discriminant functions. As shown in Fig 6, function 1 explained 61.4% of the variance and function 2 explained 38.6% of the variance; therefore, the total of variance explained by these two functions was 100%. Different *Dendrobium* species were clumped in different regions of the space. The *D*. *huoshanense* samples were entirely separated from *D*. *officinale* and *D*. *moniliforme*. *D*. *officinale* samples and *D*. *moniliforme* samples were a little closer but there was no overlap among them. These results confirmed the applicability of multi-elemental analysis for identifying different *Dendrobium* species.

## Conclusions

This study provides a detailed report on polysaccharide, total alkaloid, amino acids and trace elements in different growth years of three kinds of *Dendrobium* species and evaluated the safety of trace elements in *Dendrobium*. According to the results, the quality of *D*. *huoshanense* is the best among the three *Dendrobium* species, followed by *D*. *officinale* and *D*. *moniliforme*. Discriminant analysis was carried out on data showing the concentration of elements found in *Dendrobium* in order to classify them according to several factors. This information can be useful to address the nutritional value and safety concerns related to the use of this plant as food or medicine. This developed method may be useful to identify substitution or adulteration in commercial *Dendrobium* samples.
